# Adult neurogenesis and aging mechanisms: a collection of insights

**DOI:** 10.1038/s41598-023-45452-1

**Published:** 2023-10-23

**Authors:** Giuseppe Lupo

**Affiliations:** Department of Biology and Biotechnologies Charles Darwin, Sapienza University of Rome, 00185 Rome, Italy

**Keywords:** Neural stem cells, Ageing

## Abstract

Adult neurogenesis supports important brain functions, such as learning and memory, and its decrease during aging may contribute to the cognitive decline of the aged brain. This Collection provides insight into the role, regulation, and age-associated reduction of adult neurogenesis, by gathering studies investigating this process in different model systems, ages, and brain regions.

Adult neurogenesis is well established in rodents, where the integration of adult-born neurons into neural networks contributes to brain plasticity. In these animals, the main adult neurogenic sites are the ventricular-subventricular zone (V-SVZ) of the lateral ventricles, and the subgranular zone (SGZ) in the hippocampal dentate gyrus. These regions harbour neural stem/progenitor cells (NSPCs) that generate neuroblasts undergoing neuronal differentiation. V-SVZ neurogenesis provides new interneurons to the olfactory bulb, which contribute to olfactory functions^1^. SGZ neurogenesis supplies new granular neurons to the dentate gyrus, participating to hippocampal-dependent memory^2^.

Two features of rodent adult neurogenesis have sparked interest from a translational point of view. Firstly, mouse aging is associated with a neurogenic decrease correlating with cognitive decline^3,4^. Secondly, adult neurogenesis in mice can be modulated by external stimuli that affect cognitive performance, such as diet and physical exercise^5,6^. Nonetheless, the existence and therapeutic potential of adult neurogenesis in humans remain controversial^7,8^. Further research on adult neurogenesis is needed in different animals, brain regions, and physiopathological conditions; this Collection contributes to this effort by providing an all-round view of this process spanning its molecular mechanisms, its regulation by different stimuli and signals, and its role in brain function and aging.

The hippocampus is a crucial cognitive structure, which makes the SGZ the most fashionable paradigm to investigate adult neurogenesis. Accordingly, mouse SGZ neurogenesis is the focus of several articles in this Collection. To address the links between hippocampal neurogenesis and function Sippel et al. exploit cyclin D2-deficient mice, in which the SGZ progenitor pool is depleted, to show that the abrogation of hippocampal neurogenesis is associated with altered sleep patterns during a spatial learning test, which correlate with the decreased performance of the mutant mice^9^. These observations suggest that hippocampal neurogenesis is key to sleep-dependent memory consolidation through the modulation of sleep patterns. Since sleep disturbances increase with age, this study also provides a potential mechanism linking the age-associated decrease of neurogenesis and cognitive decline.

Exploring the molecular networks regulating NSPC self-renewal and lineage progression, Hourigan et al. uncover a new regulator of SGZ neurogenesis, the transcriptional repressor Capicua (CIC), which is dynamically expressed in the neurogenic lineage, with stronger expression in NSPCs and neurons than in neuroblasts^10^. CIC deletion causes a decrease of the NSPC pool and an impaired maturation of adult-born neurons. Since mouse aging is associated with similar alterations, investigating CIC expression pattern and functional role in the SGZ of aging mice is a promising direction. Another hint to the molecular mechanisms potentially leading to neurogenic aging comes from the study of Schuele et al., which shows that diacylglycerol lipase alpha (Dagla), the main cannabinoid (CB)-producing enzyme, is expressed in SGZ NSPCs and, at higher levels, in granular neurons^11^. Dagla deletion in NSPCs and astrocytes, but not in neurons, impaires SGZ neurogenesis, suggesting that autocrine CB signaling in NSPCs may be required for their proliferation and/or lineage progression. As with NSPC activity and neurogenesis, CB levels are also affected by age^12^; therefore, it will be interesting to investigate whether this autocrine CB-mediated mechanism is disrupted during SGZ aging.

Adult neurogenesis can be modulated by several stimuli and conditions that affect cognitive performances. Two studies in this Collection show that hippocampal neurogenesis is impaired in mouse models of intermittent hypoxia (IH), a hallmark of sleep apnea, and of type-2 diabetes mellitus (T2DM). Khuu et al. analyse the effects of IH in mice during specific stages of neurogenic lineage progression, showing that IH causes an increased pro-oxidant state that impacts neural progenitors and newborn neurons, leading to a decrease in these populations that can be rescued by exogenous antioxidants^13^. Bonds et al. analyse the hippocampal neurogenic cell populations in two different mouse models of T2DM, observing in both cases a reduction of neuroblasts and immature neurons^14^. Since IH and T2DM are associated with cognitive impairment, especially during aging, these studies have therapeutic implications.

Two other studies highlight the importance of identifying molecules capable of countering the neurogenic decline occurring during aging or disease. Arredondo et al. show that andrographolide, a plant-derived compound, can rescue the decrease in neural progenitors and neuroblasts observed in a mouse model of Alzheimer’s disease, along with the performance of these mice in a spatial memory test^15^. Hwang et al. report that lactate intake can stimulate hippocampal neurogenesis in sedentary mice, without enhancing the neurogenic increase promoted by mild exercise; however, lactate can collaborate with exercise to improve the performance in spatial memory tests, and to increase the hippocampal levels of proteins implicated in neuronal plasticity^16^.

The V-SVZ is the main neurogenic site in adult rodents based on the size of its neuronal output, but its neurogenic activity in other mammals is unclear^17^. Tepper et al. describe the age-associated changes in olfactory discrimination and in olfactory bulb immature neurons in a marsupial species, the opossum, comparing these changes in female and male animals^18^. Although both sexes show a decreased density of immature neurons in the aged olfactory bulb, this effect is more pronounced in females, which correlates with the reduced performance in an olfactory discrimination test specifically in aged females. This study supports the idea that olfactory functions rely on adult neurogenesis in mammals and suggests that sex-dependent hormonal changes significantly affect this process. Bitar et al. investigate the human V-SVZ across the lifespan through the lens of transcriptomic analyses^19^. They show that neurogenesis-related genes are sharply downregulated during infancy, whereas genes associated with the immune system increase their expression with age. These results support evidence that the human V-SVZ becomes quiescent early in life^17^, pointing to neuroinflammation as the main driver of this quiescence, as proposed in mice^20^. Whether human V-SVZ neurogenesis might be activated by targeting some of these age-modulated genes is an interesting question for future research.

Adult neurogenesis is also present in the mouse hypothalamus, although at lower levels compared to the V-SVZ and the SGZ^21^. Engel et al. follow up previous work showing that unsaturated fatty acids stimulate hypothalamic neurogenesis via the fatty acid receptor GPR40, by uncovering a role for p38 kinase and brain-derived neurotrophic factor in mediating the effects of GPR40 activation^22^. These findings may help to replace neurons with key homeostatic functions that are damaged in aging and obesity. The ability to stimulate endogenous neurogenesis may also aid the replacement of the mesencephalic dopaminergic neurons that are lost in Parkinson’s disease. Brown et al. demonstrate adult dopaminergic neurogenesis in zebrafish and its decrease during aging and in the absence of Pink1, whose mutations cause early onset Parkinson’s disease^23^. Pink1-deficiency also impairs dopaminergic neurogenesis in human midbrain organoids, suggesting that the mechanisms supporting adult dopaminergic neurogenesis in zebrafish may be clinically relevant.

Finally, in those brain regions devoid of neurogenic activity, gliogenic progenitors might be amenable to reprogramming towards neuronal fates. Sanchez-Gonzalez et al. describe the adult progeny of oligodendrocyte precursors, also known as NG2-cells, of the early postnatal mouse cerebral cortex^24^. They show that NG2-cells are a heterogeneous proliferative population, which is capable of clonal expansion and dispersion depending on age and location. Although NG2-cells generate oligodendrocytes and astrocytes, but not neurons, in physiological conditions, their reprogramming might allow neuronal repair in the cerebral cortex.

Altogether, this Collection highlights the importance of adult neurogenesis in the context of brain plasticity and its age-associated decline, and the outstanding possibilities that this research field offers for translational studies.
